# Molecular detection of porcine reproductive and respiratory syndrome virus, porcine circovirus 2 and hepatitis E virus in oral fluid compared to their detection in faeces and serum

**DOI:** 10.1186/s12917-020-02378-4

**Published:** 2020-05-27

**Authors:** Jan Plut, Urska Jamnikar-Ciglenecki, Marina Stukelj

**Affiliations:** 1 Clinic for Ruminants and Pigs, Clinic for Reproduction and Farm Animals, Veterinary Faculty University of Ljubljana, Ljubljana, Slovenia; 2 Institute of Food Safety, Feed and Environment, Veterinary Faculty University of Ljubljana, Ljubljana, Slovenia

**Keywords:** Pig oral fluid, Faeces, Serum, PRRS, Hepatitis E virus, PCV2

## Abstract

**Background:**

Porcine Reproductive and Respiratory Syndrome Virus (PRRSV), Porcine Circovirus Type 2 (PCV2) and Hepatitis E virus (HEV) are common and economically important viral disease causative agents detected in pig oral fluid (OF), faeces and serum at some infection stages. The purpose of this study was to detect PRRSV, PCV2 and HEV on six pig farms to determine which of the three sample types, OF, faeces or serum is appropriate for the diagnosis of these viruses in different pig categories.

The following pig categories were included: 5 weeks-old (w/o), 7 w/o, 9 w/o, 11 w/o weaners, fatteners and breeding sows. Pursuant to the preliminary detection of each pathogen at the selected farms, OF samples, faeces, serum pools and 10 individual sera were examined, using PCR, for each age category. If any of the viruses were found in pools of faeces and OF, then faeces and OF from positive farms were tested separately for each pig category. The viral nucleic acids were detected using RT-PCR, PCR and real-time RT-PCR, for PRRSV, PCV2 and HEV respectively.

**Results:**

PRRSV and HEV were detected on one farm and PCV2 on three others, positive results being more often obtained from the OF than from the faeces of the same animals. Ten individual serum samples from pigs from the same group of animals were also tested. The viruses were detected in almost all individual sera and OF in the same pig category with some exceptions: PRRSV was detected in the OF of fatteners but was absent in their sera; on Farm 2, PCV2 was detected in sera of 11 w/o pigs and fatteners but absent in group samples of their OF and, vice versa, in case of 9 w/o animals; HEV was detected in the OF of the youngest, 5 w/o weaners and absent in sera of the same age group.

**Conclusions:**

The primary finding of the study is that OF is a welfare-friendly, non-invasive and highly efficient matrix for pathogen detection, thus evidencing the usefulness of pig OF as a matrix in which each of the three viruses considered can be detected with the highest probability.

## Background

Oral fluid (OF), consisting of oral salivary gland products and mucous transudate, has been used for the detection of various pathogens and antibodies in humans and animals. Its use for detection of anti-Malta fever antibodies in humans was first described in 1909 [1]. In terms of swine disease Porcine Reproductive and Respiratory Virus (PRRSV), such antibodies were initially isolated from individual animal buccal samples in 1997 [2]. Increased utilisation of pig OF for molecular diagnosis of the pathogens was reported in 2008 by Prickett et al. [3]. Pursuant to these studies, pig OF sampling for the detection of pig disease was further acknowledged in the first comprehensive studies of methodology, including sampling procedure, containment and transport, pre-diagnostic processing of OF samples, and comparison with other samples previously used for the detection of PRRSV and PCV2. One of the main reasons for choosing OF sampling over other sampling methods is its stress-free ease of use vis à vis disease monitoring and consequent positive impact on pig welfare [4].

The use of OF analysis in pig health care has steadily progressed since its start and is increasing in importance. In the US, numbers have risen from 21,000 tested samples in 2010 to 370,000 samples in 2016 [5]. However, Slovenian pig farming and pig health care does not make full use of this diagnostic method. The Slovenian pig industry has declined markedly since entry of the country into the European Union in 2004. Production in Slovenia fell to a mere 259,000 pigs in 2018. There are only two farms with 3000 breeding animals, making pig production industries in Slovenia one of the smallest in Europe, thus meeting, through importing, almost 80% of pork product needs. Ninety-five percent of Slovenian farms are “backyard” farms with fewer than 10 breeding animals; 47.7% of all farms own just one or two animals. Our health care declarations do not provide mandatory protective vaccinations, individually necessary biosecurity measures, or tracking of some economically important infective diseases (data from www.pig333.com, Slovenia Statistical Office).

From the first detection of a limited number of pathogens in OF, mostly PRRSV [3], researchers now endeavour to expand the spectrum of important pathogens, specific antibodies against them, for example Classical Swine Fever [6] and bacteria *Lawsonia Intracellularis* [7], and other activities covering fields like proteomics [8]. Twelve scientific articles have been published in various scientific journals since 2018, excluding conference contributions, abstracts and others, all according to the NCBI database (https://www.ncbi.nlm.nih.gov/pubmed). These reports, concerning PRRSV and PCV2 in OF, are mostly related to sampling strategy, comparison of pooled and individual samples, and detection of viral nucleic acids and antibodies against virus antigens. When it comes to HEV, however, only one scientific article has been published since 2014 describing detection of HEV RNA in pig O. Further, despite the promising results obtained in that study, faeces remain the primary sample of interest regarding live pigs [9].

PRRSV, PCV2 and HEV are all important viral disease causative agents. Infections by PRRSV and PCV2 are common diseases and affect exclusively pigs. They account for huge economic losses [10–12]. HEV is potentially lethal for certain populations of humans in terms of chronic hepatitis, and contaminated pork and meat products are potential sources of human infection, especially HEV genotype 3, which is present predominantly in Europe [13, 14]. Individual serum samples are still most frequently used for the detection of most pig diseases in herds. Because drawing blood is technically demanding, bloody, potentially stressful, and, sometimes, fatal for pigs, other samples, including OF, are becoming increasingly popular [15]. The most likely natural route of infection for all three viruses under field conditions is the nasal-oral route, meaning that agents are present in OF at disease stages. However, the pathogenesis of these diseases differs significantly. Further, detection of the virus’ genetic material for molecular diagnostics is only possible for a limited amount of time. This makes the choice of an appropriate sampling method challenging. PRRSV and PCV2, for example, involve long-lasting recurring viremic periods. When PRRS viremia starts in the suckling period it can last for more than 60 days, i.e. throughout the whole weaning period. It lasts 35–42 days in fatteners, up to 1 week in breeding pigs for PRRS, and up to 70 days for PCV2 in post-weaning and, later, during fattening [16, 17]. In terms of HEV, viremia can be short-lasting, even absent [18], necessitating use of alternative sampling for detection in vivo, OF being amongst them. One characteristic for all these diseases is that they can be detected in different organ systems after the elimination of circulating virus. This can be used to advantage when samples other than sera are used for analysis [9, 17, 19]. Using some of this information, the purpose of this study was to detect PRRSV, PCV2 and HEV in different pig enterprises to determine which of these samples, OF, faeces or serum, is the most appropriate for the diagnosis of these viruses in different pig categories.

## Results

Pooled samples of OF and faeces from all pig categories from each farm were tested (10 μL of each eluent) for the preliminary detection of each of the three pathogens, in order to determine whether farms were either positive or negative in terms of PCR Reaction (Table 1).

**Table 1:** Preliminary testing of pooled samples of OF and faeces for each farm

			*Farm*					
			1	2	3	4	5	6
*Selected pathogens*	PRRSV^a^	OF	*neg.*	*neg.*	*neg.*	*neg.*	*pos.*	*neg.*
		faeces	*neg.*	*neg.*	*neg.*	*neg.*	*neg.*	*neg.*
	PCV2^b^	OF	*neg.*	*pos.*	*neg.*	*pos.*	*neg.*	*pos.*
		faeces	*neg.*	*pos.*	*neg.*	*neg.*	*neg.*	*pos.*
	HEV^c^	OF	*neg.*	*neg.*	*neg.*	*neg.*	*neg.*	*pos.*
		faeces	*neg.*	*neg.*	*neg.*	*neg.*	*neg.*	*pos.*

*neg*. negative result, *pos.* positive result

^a^classic RT-PCR; ^b^classic PCR; ^c^real-time RT-PCR

The pooled group samples from farms 1 and 3 were negative for all three pathogens, while all the samples from the other farms were positive for at least one pathogen in OF and/or faeces (Table 1).

After isolated nucleic acid from group samples of OF and faeces, from all categories, had been pooled, a preliminary classification of the farm was made to determine the presence of each of the three types of virus.

After pooled samples were determined to be positive, samples from each category were tested individually to determine in which category of pigs the pathogen was to be found, as well as if the pathogen can be discovered in all three different samples with PCR (Table 2). If any of the viruses were detected in OF or faeces, all 10 sera samples from the same pig group were tested individually for the same virus. If both OF and faeces tested negative, two pools of five sera were tested for all three pathogens to confirm the initial result; if serum pools were also negative, individual sera samples were not tested (NT).

**Table 2:** Presence of viruses in OF, faeces and serum on each farm in different pig categories

	Farm	5 w/o	7 w/o	9 w/o	11 w/o	Fatteners	Breeding sows
PRRSV^a^							
OF	5	*neg.*	*pos.*	*pos.*	*pos.*	*pos.*	*neg.*
faeces		*neg.*	*neg.*	*neg.*	*pos.*	*neg.*	*neg.*
serum pool		*neg.*	*NT*	*NT*	*NT*	*NT*	*neg.*
individual sera^d^		*NT*	10/10	10/10	10/10	0/10	*NT*
PCV2^b^							
OF	2	*neg.*	*neg.*	*pos.*	*neg.*	*neg.*	*neg.*
faeces		*neg.*	*neg.*	*pos.*	*neg.*	*neg.*	*neg.*
serum pool		*neg*.	*neg.*	*neg.*	*pos.*	*pos*.	*neg.*
individual sera^d^		*NT*	*NT*	0/10	8/10	3/10	*NT*
OF	4	*neg.*	*neg.*	*neg.*	*pos.*	*neg.*	*neg.*
faeces		*neg.*	*neg.*	*neg.*	*neg.*	*neg.*	*neg.*
serum pool		*neg.*	*neg.*	*pos.*	*neg.*	*neg.*	*neg.*
individual sera^d^		*NT*	*NT*	6/10	0/10	*NT*	*NT*
OF	6	*pos.*	*pos.*	*pos.*	*neg.*	*neg.*	*neg.*
faeces		*pos.*	*pos.*	*pos.*	*neg.*	*neg.*	*neg.*
serum pool		*NT*	*NT*	*NT*	*neg.*	*neg.*	*neg.*
individual sera^d^		2/10	3/10	9/10	*NT*	*NT*	*NT*
HEV^c^							
OF	6	*pos.*	*pos.*	*pos.*	*neg.*	*neg.*	*neg.*
faeces		*pos.*	*pos.*	*pos.*	*neg.*	*neg.*	*neg.*
serum pool		*NT*	*NT*	*NT*	*neg*.	*neg*.	*neg*.
individual sera^d^		0/10	1/10	2/10	*NT*	*NT*	*NT*

*neg.* negative result, *pos.* positive result, *NT* not tested

^a^ classic RT-PCR

^b^ classic PCR

^c^ real-time RT-PCR

^d^ten individual sera were tested from each pig age group. Results are shown pos./all tested sera

PRRSV was discovered on Farm 5; OF samples were deemed positive by RT-PCR for 7, 9 and 11 w/o weaners and fatteners (estimated 15 w/o), while all faecal samples, except the one from 11 w/o weaners, were negative. PRRSV RNA was detected in all weaner serum samples, while all samples from fatteners were negative.

Farms 2, 4 and 6 were PCV2 positive. Viral DNA could only be detected in OF and faeces in weaners. OF and faeces of fatteners and breeding sows were all negative. On Farms 2 and 6, viral DNA was detected in OF and faeces samples from the same category but, on Farm 4, only OF from 11 w/o weaners was positive, and faeces negative, shown by classic PCR. Viral DNA was found in sera of different pig categories of all three PCV2 positive farms. Only on Farm 6 the viral DNA could be found in all three tested samples of the same pig category. On Farm 2 OF and faeces of 9 w/o weaners were positive and sera were negative for PCV2 DNA, but in 11 w/o weaners and fatteners, the situation was reversed; their OF and faeces were negative and their sera were positive. The similar trend was observed in 9 w/o and 11 w/o weaners on Farm 4, when comparing group samples to sera.

HEV was detected in the youngest weaners, aged 5, 7 and 9 weeks old, on Farm 6; both OF and faeces samples tested positive, whilst only one serum sample from 7 w/o weaners and two from 9 w/o weaners were deemed HEV RNA positive, using quantitative RT-PCR.

Samples were compared using Fisher’s exact test, which evidenced strong correlation between faecal and OF samples when determining PCV2 (*p* = 0.001). For other samples, correlation could not be characterized as statistically significant.

## Discussion

Pig OF has been regarded for some years as one of the most appropriate samples for pathogen detection [3, 20, 21]. Major research in this field started towards the end of the previous decade with the goal of finding a way to make sample collection less stressful for pigs, and easier, faster and cheaper for those performing sampling [4, 22]. Previous studies provided some insight into OF collection, pre-diagnostic procedures and results regarding different pathogens; results were mostly promising; nevertheless, unanswered questions regarding methodology remain, including incomplete standardised procedures in terms of pre-diagnosis and the variability of its results when compared to those of other individual or group samples, e.g. serum, nose swabs, post-mortem isolation from organs, and group faeces samples [23, 24].

In our study, group OF samples and group faecal samples were compared with individual serum samples from different pig categories from six Slovenian farms, each with different numbers of disease history data. PRRSV, PCV2 and HEV were chosen since these viruses represent quite common pathogens in the pig industry and are either economically important for pig health, or constitute a possible food safety threat, such as HEV. All diseases have their own pathophysiological characteristics and, therefore, are detectable in a variety of infection stage samples. Even though OF samples were not taken from individual pigs, results show they are of great use for determining viral presence on farms regarding the diseases considered. Group sampling in the case of OF is seen to be more effective than the use of group faecal samples, probably due to the presence of greater amounts of inhibitory substances in faeces [23–25]. Statistically significant correlation between OF and faeces samples was shown with more than 95% probability (*p* = 0.001); in other cases, correlation was disputed by a Fisher’s test value of *p* < 0.05, although the results favoured OF over other samples in terms of PRRS and HEV detection. Compared with individual serum samples, OF showed complementary results. Variation was noted in PCV2 DNA detection. OF samples were collected from a relatively small number of pigs compared to the study by Nielsen et al. in 2018 [20] (a maximum up to 20 pigs in a group pen). OF was sampled pursuant to all pigs being observed chewing the ropes for collecting OF. Faeces from the rectum of the animals in the pen were added directly to containers to ensure that samples were representative. Even though sensitivity fell by an estimated 27–100% when five sera samples were pooled, as when compared to individual samples, our results are of great value, especially in terms of studies of prevalence [26].

When considering PRRSV, PCV2 and HEV, several studies have investigated simultaneous co-infection with two viruses. This took place on Farm 6, where PCV2 and HEV were found in weaning pigs of all ages. Salines et al. described the same virus combination in 2019, finding a statistically significant concentration of HEV in faeces during infection. Seroconversion for anti-HEV antibodies took longer and the transmission rate for HEV was approximately three times higher in co-infected pigs [27]. Another study by the same author states that the presence of immunosuppressive virus increases the viral concentration of HEV in the liver at slaughter [28]. Accordingly, the fact that the degree of infection by HEV on Farm 6 was high, could be due to PCV2 coinfection which led to modification of immune system caused by the immunosuppressive effect of PCV2. This was harder to assess because our primary focus was co-infection and our environment was uncontrolled. But looking from a viewpoint opposite to that of Salines, PCV2 was detected in weaner’s sera and, generally, in more categories than on the other two farms. The farm owner stated that he experienced trouble with a higher mortality rate, poor average daily gain and wasting during this stage. Yang et al. in 2015 provided evidence for fatalities in weaned pigs co-infected with PCV2 and HEV [29]. HEV is usually depicted as benign and not as a swine pathogen, but it may be part of a multifactor clinical outbreak catalyst, thus negatively affecting pork quality.

PRRSV was only found on Farm 5, a farm that had previously experienced disease outbreak. Even though this farm acclimates its gilts before transferring them to the breeding herd, transmission to offspring still occurs. Youngest weaners are disease-free due to immunity provided by colostral; the oldest get infected. Viral RNA was present in OF and sera tested positive for weaners aged 7, 9 and 11 weeks. PRRSV RNA was only found in the faeces of 11 w/o weaners, as also evidenced in study by Christianson et al. (1993) where PRRSV only appears in faeces intermittently [30]. Fatteners appear to eliminate virus from sera, presumably due to the appearance of antibodies in the sera; even so, the virus appears to persist in OF for longer periods of time [19]. This means that OF can be a sample of choice for diagnostic use, especially for longer periods following infection, that is, during the weaning-fattening period and for replacement of gilts before entering the breeding herd, when the virus is eliminated from the bloodstream, but present in other tissue.

Although PCV2 is considered to be ubiquitous, its presence was only detected on half the farms concerned, of which only one used anti-PCV2 vaccination as part of their preventive program. On Farm 2, where pregnant sows were vaccinated against PCV2 with a commercially-sourced vaccine, none of the younger weaners were viremic. The virus was found in the OF and faeces of 9 w/o weaners. However, the situation in the case of 11 w/o weaners was different, since PCV2 was not found in OF and faeces. This virus was present in 80% (11 w/o weaners) and 30% (fatteners) of pig sera. It appears that the immunity acquired by means of preventive programs varies between 9 and 11 w/o and, as the virus is still present in the environment, weaners and fatteners become viremic. In the case of Farm 2, it appears as if their prevention programme does not completely protect pigs from the viremic phase; viral DNA could be detected later in OF and faeces of 9 w/o and in sera of 11 w/o weaners and of fattening pigs. Feng et al. (2016) suggest that 3 w/o piglets should also be vaccinated also, since it extends protection against the pathogen until pigs are up to 25 weeks old, regardless of the presence of maternally-derived antibody [31], and that protocol of vaccination against PCV2 has since been applied on many Slovenian farms as a preventive measure.

PCV2 was also detected on Farm 4 despite the fact that a local veterinarian stated that this virus had never before been detected in any samples taken from pigs in this farm. These pigs were, otherwise, not vaccinated. PCV2 was only detected in the sera of 60% of 9 w/o weaners, and not in OF. This situation was reversed for 11 w/o weaners, which could mean that pigs become infected by PCV2 at around 9 w/o and eliminate the virus from their bloodstreams in a short period of time. This was in accordance with a study by Grau-Roma et al. (2007). Pigs are protected by maternal immunity until week 8 and then by blood viral load peaks when 10 w/o; from then on, the virus is eliminated from the bloodstream and then from the organism [32]. Nonetheless, OF appears to be a good diagnostic tool for determining farm prevalence. Research by Nielsen et al. stated that it is even better than serum [20].

On Farm 6, PCV2 was detected in the youngest categories of weaners (5, 7 and 9 w/o). Pigs on this farm had not been vaccinated. PCV2 was detected in both faeces and OF, and in sera. Our statistical analysis indicates that OF is more effective than faeces are for detecting viruses (*p* = 0.001). The infection timeline and viremia onset are in accordance with previous studies: most pigs were infected at 4–11 w/o and repetitive viremia is present from day 7 to day 70 [32, 33]. Farm 6 was, in addition to being PCV2-positive, the only HEV-positive farm, despite the latter virus being described as ubiquitous [34]. HEV DNA was shown, using RT-PCR, to be present in OF and faeces of the three youngest categories of weaners. The results show that disease is absent in older categories, in accordance with previous reports: pigs become infected at around 2–3 months of age and this persists in some excrement for 3–7 weeks. If viremia appears, it is usually present for short periods of time, between one and 2 weeks [18, 34]. None of the weaners were viremic by week 5; one in ten were viremic at 7 weeks, and two in ten at week 9, which indicates that HEV only spreads sporadically into the bloodstream. Although the virus is supposed to replicate in the lower gastrointestinal tract [35], OF concentration is apparently high enough for molecular detection. Compared to group faeces samples, OF is collected more easily and all pigs from the group chew on the ropes. Faeces for collection are not evenly distributed over the pens’ floors: some excrement will be old, some will fall through floors’ slats. If faeces are obtained directly from the rectum of animals, the procedure can be time-consuming and stressful for pigs. As mentioned earlier, statistical analysis supports the usefulness of pig OF for detecting PCV2 DNA, but neither proved nor disproved correlation in cases of HEV RNA detection in OF, faeces or serum due to the low number of positive samples in our study.

## Conclusion

This study provides evidence that OF is an effective matrix for the detection of PRRSV, PCV2 and HEV nucleic acids. In live pigs, serum samples are usually tested for the presence of PRRSV and PCV2, and faeces for HEV presence. Our study shows that the same pathogens can be detected by means of OF with equal or even greater certainty. OF is as good as, or even better for detection of PRRSV and HEV than individual serum samples and faeces. OF was statistically more effective than faeces (*p* = 0.001) for detecting PCV2, but the results were not completely complementary with those from serum detection.

## Methods

### Animals and farms

Samples were taken at six pig farms: one small, two-site farm (Farm 1), with approximately 500 breeding sows, three small one-site farms with less than 100 breeding sows (Farms 2, 3 and 6), and two large one-site farms (Farms 4 and 5) with more than 1000 breeding sows. The smallest three farms in our study do not quarantine before gilt replacement, but farms 2 and 6 confirm that newly bought animals are PRRS antibody negative. Epidemiological data for Farm 3 were not known, and preventive vaccination against PCV2 or PRRS is not implied. Animals were placed in groups of ten individual pigs in separate crates and divided into six age-dependent categories at all farms: 5 weeks-old (w/o); 7 w/o; 9 w/o; 11 w/o weaners; fatteners; and breeding sows. Vaccination against PCV2 is part of Farm 2’s preventive protocol. No on-site preventive measures are applied against our selected pathogens in other farms. No ethical approval by the Slovenian Ministry of Agriculture, Forestry and Food’s Administration of the Republic of Slovenia for Food Safety, Veterinary Sector and Plant Protection was needed for the purposes of this study.

### Samples

Ten individual blood samples were drawn from each pig category, from each of the 6 farms. A total of 360 individual blood samples were collected from the anterior vena cava. Group OF samples were obtained by means of the cotton ropes provided in the IDEXX Oral Fluid Collection Kit. Undyed-Cotton 3-Strand Twisted Rope was hung for half-an-hour above an open spot in the middle of pens, away from feed and drinking water. After this, the rope was removed and OF squeezed from it into sterile 50 ml screw cap plastic containers. Group samples of fresh faeces were collected from these pigs also, at random pen sites, and a smaller amount directly from recta, and put into 100 ml sterile screw cap plastic containers. A total of 36 OF samples and 36 faeces samples were collected and examined from each of the six animal categories on each of the six farms. Group blood samples were also drawn from the same groups of pigs.

Samples were transported to the laboratory in a refrigerated box at 4 °C and immediately treated as follows. OF samples were centrifuged for 10 min at 2000×*g* and supernatant was stored at − 70 °C. A 10% suspension in RPMI-1640 (Thermo Fisher Scientific, Carlsbad, CA, USA) was prepared from each faecal sample. Suspensions were centrifuged at 2000×*g* for 10 min before the supernatant was transferred to sterile 20 ml screw cap plastic containers and stored at − 70 °C for further testing. After coagula formation, sera were centrifuged for 10 min at 3000×*g*. These samples were stored individually in 20 ml sterile cryotubes at − 70 °C. 10 μL of OF eluent and faeces samples from each of the six pig categories were taken and pooled for preliminary determination as to whether the animals on the farms were virus-positive. If any of the viruses were found in these pools, then faeces and OF from positive farms were tested separately for each pig category. Ten individual serum samples were obtained from pigs from each category on every farm. If any of the viruses were detected in OF or faeces, all 10 sera samples from the same pig group were tested individually for the same virus; if both OF and faeces tested negative, two pools of 5 sera (140 μL) were tested for all three pathogens to confirm the initial result; if serum pools were also negative, individual sera samples were not tested. Following this protocol, 150 individual serum samples were tested for different pathogens.

### DNA/RNA extraction

DNA and RNA samples were extracted manually using the QIAamp Viral RNA Mini Kit (Qiagen, Germany), according to the manufacturer’s instructions. Accordingly, nucleic acids were extracted from 140 μL of the supernatant and eluted in 60 μL of elution buffer.

### RT-PCR for PRRS detection

RT-PCR, by means of the One-Step RT-PCR Kit (Qiagen, Germany) and specific primers (Table 3), was performed to specifically detect PRRSV. The final reaction volume of 25 μL comprised 5 μL of 5x PCR buffer, 11 μL DNase/RNase-free water, 1 μL of 10 mM dNTP mix, 0.5 μL of each primer (20 pmol/μL) (Table 3), 1 μL RT-PCR Enzyme Mix, and 6 μL of RNA. Amplification was performed by means of the Mastercycler Nexus Gradient (Eppendorf, Germany) under thermocycling conditions of 30 min at 50 °C and 15 min at 94 °C, followed by 40 cycles of denaturation at 94 °C for 30 s, annealing at 60 °C for 30 s, elongation at 72 °C for 14 min and, finally, followed by elongation at 72 °C for 10 min. PCR products were visualized on 1.8% (w/v) agarose gel according to the expected size of the PCR product (Table 3).

**Table 3:** Specific primers and probes used in this study

Detected virus	Name	Primer and probe sequence (5′-3′)	PCR product	Reference
PRRSV	F-P1	CCA GCC AGT CAA TCA RCT GTG	291 bp	Donadeu et al., 1999 [36]
	R-P2	GCG AAT CAG GCG CAC WGT ATG		
PCV2	2A	CAC CTT CGG ATA TAC TGT CAA	501 bp	Grierson et al., 2004 [37]
	2B	TAC ATG GTT ACA CGG ATA TTG TA		
HEV	JVHEV-F	GGT GGT TTC TGG GGT GAC	/	Jothikumar et al., 2006 [38]
	JVHEV-R	AGG GGT TGG TTG GAT GAA		
	JVHEV-P	6-FAM-TGA TTC TCA GCC CTT CGC-BMQ		

### PCR for PCV2 detection

Platinum PCR SuperMix (Invitrogen) was used for PCV2 detection. A 25 μL reaction mixture, composed of 21.5 μL Platinum PCS SuperMix, 0.75 μL of each specific primer (Table 3), and 2 μL of DNA was used for this purpose. Mastercycler Nexus Gradient (Eppendorf, Germany) thermal conditions were 2 min at 94 °C, followed by 35 cycles of denaturation at 94 °C for 30 s, annealing at 55 °C for 30 s and elongation at 72 °C for 1 min, followed by final elongation at 72 °C for 7 min. PCR products were visualized on 1.8% (w/v) agarose gel according to the expected size of the PCR product (Table 3).

### Real-time RT-PCR for HEV detection

Real-time RT-PCR was performed by means of the SuperScript III Platinum One-Step Quantitative RT-PCR System (Thermofisher Scientific). For HEV detection, a 25 μL final reaction mixture composed of 12.5 μL 2x reaction mix, 0.5 μL 50 mM MgSO_4_, 0.5 μL ROX Reference Dye (1:10), 5 μL DNase/RNase-free water, 0.75 μL of each primer (20 pmol/μL), 0.5 μL of specific probe (5 pmol/μL) (Table 3), 0.5 μL SuperScript® III/Platinum® *Taq* Mix, and 4 μL of RNA was used. The reaction was performed by means of QuantStudio3 (Thermo Fisher Scientific, Massachusetts, USA) under thermocycling conditions of 15 min at 50 °C and 10 min at 95 °C, followed by 45 cycles of denaturation at 95 °C for 15 s, then annealing at 55 °C for 1 min.

### Statistical analysis

Test results are presented, together with basic descriptive statistics. For each disease agent and for matrix used for detection of them, the results of testing of each different type of samples were compared with results of the others, using Fisher’s exact test. The analysis was performed using R Statistical Software, version 3.6.0 (Foundation for Statistical Computing, Vienna, Austria); and *P* values less than 0.05 considered to be statistically significant.

## Data Availability

The datasets used and/or analyzed during the current study are available from the corresponding author on reasonable request.

## Abbreviations

HEV: Hepatitis E virus
OF: Oral fluid
PCR: Polymerase chain reaction
PCV2: Porcine circovirus 2
PRRSV: Porcine reproductive and respiratory virus
RT-PCR: Reverse transcriptase-polymerase chain reaction
